# Molecular Basis of Non-β-Lactam Antibiotics Resistance in *Staphylococcus aureus*

**DOI:** 10.3390/antibiotics11101378

**Published:** 2022-10-08

**Authors:** Harshad Lade, Hwang-Soo Joo, Jae-Seok Kim

**Affiliations:** 1 Department of Laboratory Medicine, Hallym University College of Medicine, Kangdong Sacred Heart Hospital, Seoul 05355, Korea; 2 Department of Biotechnology, College of Engineering, Duksung Women’s University, Seoul 01369, Korea

**Keywords:** *Staphylococcus aureus*, MRSA, antimicrobial resistance, molecular basis, macrolides, glycopeptides, lipopeptides

## Abstract

Methicillin-resistant *Staphylococcus aureus* (MRSA) is one of the most successful human pathogens with the potential to cause significant morbidity and mortality. MRSA has acquired resistance to almost all β-lactam antibiotics, including the new-generation cephalosporins, and is often also resistant to multiple other antibiotic classes. The expression of penicillin-binding protein 2a (PBP2a) is the primary basis for β-lactams resistance by MRSA, but it is coupled with other resistance mechanisms, conferring resistance to non-β-lactam antibiotics. The multiplicity of resistance mechanisms includes target modification, enzymatic drug inactivation, and decreased antibiotic uptake or efflux. This review highlights the molecular basis of resistance to non-β-lactam antibiotics recommended to treat MRSA infections such as macrolides, lincosamides, aminoglycosides, glycopeptides, oxazolidinones, lipopeptides, and others. A thorough understanding of the molecular and biochemical basis of antibiotic resistance in clinical isolates could help in developing promising therapies and molecular detection methods of antibiotic resistance.

## 1. Antibiotic Resistance and Human Health Risk

**Figure 1:**
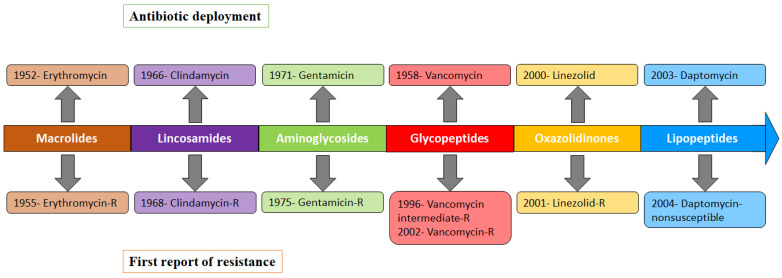
Timeline of the key non-β-lactam antibiotics deployment and the first reported cases of *S. aureus* resistance identified.

The discovery of penicillin by Alexander Fleming in 1928 was one of the largest triumphs of biomedical research [1], and its introduction for clinical use in 1943 began a new era in the treatment of bacterial infection. Alexander Fleming won a Nobel Prize for the discovery of penicillin in 1945, and during his lecture, he warned that overuse of antibiotics could result in selection for resistant bacteria. True to this prediction, the extensive use of antibiotics has led to the selection and expansion of penicillin-resistant bacteria. In 1940, even before the introduction of penicillin for clinical practice, Abraham and Chain identified an enzyme (penicillinase) from *Escherichia coli* able to destroy penicillin [2]. Following the development of penicillin, multiple classes of antibiotics were developed and launched to treat bacterial infections: macrolides, e.g., erythromycin; lincosamides, e.g., clindamycin; aminoglycosides, e.g., gentamicin; glycopeptides, e.g., vancomycin; oxazolidinones, e.g., linezolid; lipopeptides, e.g., daptomycin; tetracyclines, e.g., tetracycline; fluoroquinolones, e.g., ciprofloxacin; pyrimidines/sulfonamides, e.g., trimethoprim–sulfamethoxazole, and others (Figure 1). Unfortunately, many bacterial pathogens associated with epidemics of human diseases have evolved resistance to almost every sequential antibiotic introduced to target it. Here, the emergence of non-β-lactam antibiotics resistance is exemplified in the bacterial pathogen *Staphylococcus aureus,* which causes a wide range of infectious diseases.

Antimicrobial resistance (AMR) has been observed in most bacteria but is particularly problematic in hospital-acquired infections from multidrug-resistant ESKAPE (*Enterococcus faecium*, *Staphylococcus aureus*, *Klebsiella pneumoniae*, *Acinetobacter baumannii, Pseudomonas aeruginosa*, and *Enterobacter* species) pathogens [3,4]. The ESKAPE pathogens are capable of escaping the bactericidal action of antibiotics and represent the paradigms for resistance, pathogenesis, and disease transmission in both hospital and community settings [3,5]. The emergence of antibiotic-resistant bacteria causing infectious diseases is a serious public health concern [6]. The World Health Organization (WHO) has described antibiotic resistance as one of the serious threats to global public health, food security, and development today [7]. A 2019 joint report by the United Nations (UN), World Health Organization (WHO), and World Organization for Animal Health (WOAH) states that if no action is taken, drug-resistant diseases could cause 10 million deaths worldwide each year by 2050 with more than $100 trillion economic output loss [8]. The U.S. Centers for Disease Control and Prevention (CDC) estimates that more than 2.8 million infections and 35,000 deaths occur due to antimicrobial-resistant pathogens every year in the United States, with this number expected to rise as more antimicrobial-resistant strains evolve [9]. The rise in antibiotic resistance is of concern in *S. aureus*, which has acquired resistance to almost every sequential antibiotic introduced to target it. For instance, an estimated 323,700 cases of methicillin-resistant *S. aureus* (MRSA) infections in hospitalized patients with 10,600 death were reported in the United States in 2017 [10]. The spread of MRSA has emerged as a global health concern because infections with MRSA are associated with significant morbidity and mortality.

## 2. Emergence of Methicillin-Resistant *Staphylococcus aureus* (MRSA)

The infectious diseases caused by *S. aureus* were well-treated by penicillin in the 1940s, but with the widespread use of this agent, penicillin-resistant *S. aureus* began to appear in the clinic. The first penicillin-resistant *S. aureus* infection was reported in 1942 [11], and a penicillinase from *S. aureus* that destroys penicillin was identified in 1944 by Kirby [12]. To combat penicillin-resistant *S. aureus* infection, methicillin (celbenin), semisynthetic β-lactamase-resistant penicillin was introduced to clinical practice in the United Kingdom in 1959 [13]. In 1961, soon after the introduction of methicillin, MRSA strains were identified among clinical isolates from patients hospitalized in the United Kingdom by Patricia Jevons [14]. Between the first reports of MRSA observed in 1961 and the 1990s, infection was common in healthcare settings (HA-MRSA) [15]. However, by the 1990s, MRSA infections has rapidly spread in the community (CA-MRSA) [16]. MRSA is one of the major causes of hospital-acquired infection globally and also occurs outside and independent of hospitals by CA-MRSA [17] and, since the mid-2000s, by livestock-associated MRSA (LA-MRSA) [18]. Several *S. aureus* clones (strains indistinguishable from each other by a variety of genetic tests) have developed into MRSA, which confer resistance to most β-lactam antibiotics. Furthermore, the prevalence of MRSA strains resistant to multiple non-β-lactam antibiotics has steadily increased and now become a major human health threat in infectious diseases [19]. 

Methicillin resistance is mediated by *mecA* gene [20], acquired by horizontal transfer of a mobile genetic element staphylococcal cassette chromosome *mec* (SCC*mec*) [21]. The *mecA* gene encodes an alternative penicillin-binding protein 2a that has a low affinity for β-lactam antibiotics [22,23], resulting in resistance to this entire class. PBP2a enables *S. aureus* to maintain cell wall synthesis when other PBPs are inhibited by β-lactams [24]. MRSA strains, besides being resistant to nearly all β-lactams, are often resistant to antibiotics of other classes such as macrolides, lincosamides, aminoglycosides, glycopeptides, oxazolidinones, and lipopeptides [25,26]. For example, complicated urinary tract infections (cUTIs) caused by MRSA are commonly treated with vancomycin [27], but strains with decreased susceptibility, designated as vancomycin-intermediate *S. aureus* (VISA)m emerged in 1996 [28]. Furthermore, clindamycin (discovered in 1966) has been generally used to treat skin and soft-tissue infection (SSTI) caused by CA-MRSA [29], and strains with clindamycin resistance were reported in 1968 [30]. 

## 3. Molecular Basis of Non-β-Lactams Resistance

**Figure 2:**
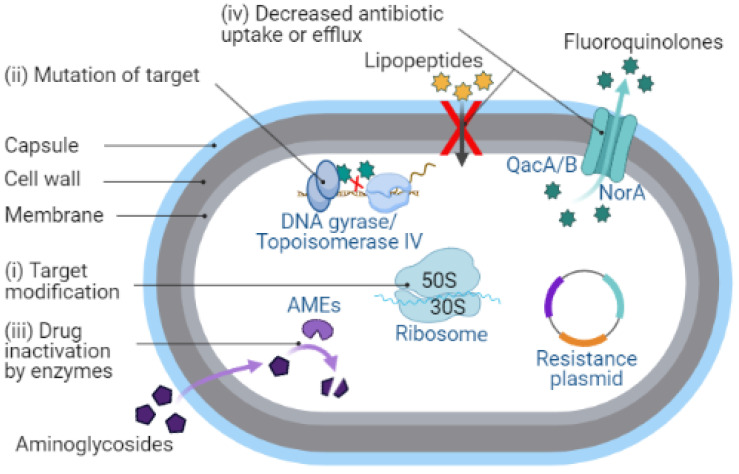
Schematic representation of the mechanisms of antibiotic resistance in *S. aureus*. (i) Target modification: change in the structure or composition of the target site in a bacterial cell can stop the antibiotic to bind, thus shielding it from the antibiotic. Modification of the bacterial ribosome by 23S ribosomal RNA (rRNA) methyltransferase (encoded by *erm* genes) leads to a conformational change in the ribosome target [31,32], thereby preventing the binding of erythromycin to the ribosomal target. (ii) Mutation of target: mutations in the target can prevent the antibiotic from binding, or if it binds, preventing it from inhibiting the target. Mutation in the DNA topoisomerase IV subunit A (encoded by *grlA* gene) and an essential enzyme DNA gyrase subunit A (encoded by *gyrA* gene) is the main contributor to fluoroquinolone resistance in *S. aureus* [33,34,35]. (iii) Drug inactivation by enzymes: several *S. aureus* enzymes modify the structure of antibiotics or break them down to make them inactive. The bifunctional aminoglycoside-modifying enzyme (AME) AAC(6′)/APH(2″) (encoded by *aac(6*′*)/aph(2*″) genes) confers resistance to aminoglycosides via acetyltransferase and phosphotransferase activities [36,37]. (iv) Decreased antibiotic uptake or efflux: decrease in the permeability of cell membrane to drugs makes it more difficult to pass through or activation of an efflux pump that removes antibiotics from the bacterial cell. The *norA, qacA*/*B,* and *smr* (*qacC/D*) genes encoding multidrug efflux pump proteins are found mainly in *S. aureus* clinical isolates and mediate resistance to fluoroquinolones, tetracyclines, and reduced susceptibility to certain antiseptics [38,39].

*S. aureus* has developed numerous mechanisms to neutralize the effect of antibiotics. Antibiotic resistance is commonly associated with the acquisition of resistance genes or mutations affecting central biochemical processes. MRSA confers resistance to non-β-lactam antibiotics by various mechanisms, such as (i) target modification, (ii) mutation of target, (iii) drug inactivation by enzymes, and (iv) decreased antibiotic uptake or efflux (Figure 2). 

### 3.1. Target Modification

Antibiotics work by binding to a cellular target so that an essential biochemical process is blocked. An alteration of the target structure prevents antibiotic binding, or it binds weakly, and thus acts as a self-resistance mechanism. The ribosome is a complex molecular machine associated with protein synthesis, and alteration of the drug-binding site through modification of rRNA results in resistance to ribosome-targeting antibiotics. For example, modification of the bacterial ribosome by 23S rRNA methyltransferase [40] prevents the binding of macrolides to ribosomal targets [31,32]. Methylation of 23S rRNA by chloramphenicol-florfenicol resistance (*cfr*) gene encoded rRNA methyltransferase alters the drug-binging site 50S ribosomal subunit [41], thus reducing the ability of chloramphenicol and clindamycin antibiotics to inhibit the ribosomes translational activity. RNA methyltransferase, the gene product of *cfr* from *S. sciuri* [42] targets nucleotide A2503 of 23S rRNA and inhibits ribose methylation at nucleotide C2498, thereby causing resistance to chloramphenicol, florfenicol, and clindamycin in *S. aureus* laboratory strain [42].

### 3.2. Mutation of Target

Chromosomal mutations that drive antibiotic resistance often arise within the genes that encode targets. Mutation of the target plays a major role in the development of resistance across distinct antibiotics such as mutations in the DNA topoisomerase IV and DNA gyrase with fluoroquinolones [33,34,35], alterations to RNA polymerase with high-level resistance to rifampicin [43], and ribosomal mutations (*tetM* and *tetO*) with tetracyclines [44,45]. Mutations in the chromosomal genes *grlA* (referred to as *parC* in other bacteria) (encoding DNA topoisomerase IV subunit A) [33,34,35,46] and *gyrA* (encoding an essential enzyme DNA gyrase subunit A) [33,34,35] are the primary mechanisms of fluoroquinolones resistance in *S. aureus*. The two enzymes are associated with the overlapping and opening of the double-stranded DNA during replication. Mutations of the *grlA* gene affect the amino acid codons Ser80, Glu84, and Ala116 of GrlA enzyme subunit [35,46], whereas *gyrA* gene mutations affect the amino acid codons Ser84, Ser85, and Glu88 of GyrA enzyme subunit [34,47] in the quinolone-resistance-determining region (QRDR). The changes in amino acids, particularly those in certain regions of each GrlA and GyrA enzyme subunit called the QRDR, decrease the binding affinity of enzymes and make them less sensitive to inhibition by fluoroquinolones. Nearly all quinolone-resistant *S. aureus* strains substitute Ser84 of GyrA with Leu or, in some other strains, Ser80 of GrlA with Phe [48,49].

### 3.3. Drug Inactivation by Enzymes

The enzymatic modification that renders antibiotics of decreased affinity for their main target 16S rRNA is the most prevalent mechanism of aminoglycosides resistance in *S. aureus* [50]. AMEs catalyze the modification at –OH or –NH2 groups of the 2-deoxystreptamine nucleus or the sugar moieties of aminoglycosides [51]. Resistance to the aminoglycoside antibiotics gentamicin, tobramycin, and kanamycin is generally mediated by a bifunctional AME AAC(6′)/APH(2″) encoded by *aac(6*′*)/aph(2*″) gene that specifies 6′-acetyltransferase [AAC(6′)] and 2″-phosphotransferase [APH(2″)] aminoglycoside-modifying activities [36,37]. Esterases encoded by *ere* genes [31,32] and phosphotransferases encoded by *mph* genes [52] confer resistance to erythromycin and other 14-, 15-, and 16-membered macrolides in *S. aureus*. Thiol-S-transferase (encoded by *fosB* gene) catalyzes the inactivation of fosfomycin [53,54], which is used to treat UTIs.

### 3.4. Decreased Antibiotic Uptake or Efflux

Resistance can develop either by decreasing the permeability of the cell membrane or by increasing the efflux of antibiotics from the cell through changes in membrane permeability [55]. NorA, QacA/B, and Smr (Staphylococcal multidrug resistance, also known as QacC/D) are multidrug efflux membrane proteins found mainly in *S. aureus* clinical isolates [56,57]. NorA (encoded by *norA* gene) is a chromosomally encoded multidrug efflux pump protein of the core genome of *S. aureus* [58] associated with resistance to fluoroquinolones, tetracyclines, and several antiseptics (chlorhexidine digluconate, cetrimide, benzalkonium chloride) [59]. The *qacA* and *qacB* genes encode an efflux pump protein that has been associated with increased resistance to fluoroquinolones and chlorhexidine tolerance [60,61,62]. Furthermore, mutations in genes encoding efflux pumps make the antibiotic export more efficient. For example, mutations in the bacterial DNA can lead the bacteria to produce more of a certain efflux pump. In staphylococci, the tetracyclines resistance is mediated by Tet efflux pumps TetA(K) and TetA(L), which are members of the major facilitator superfamily (MFS) transporters with 14 transmembrane domains. TetK is encoded by the small multicopy plasmid pT181 and is integrated within the chromosomal SCC*mec*III cassette of MRSA strains [63].

## 4. MRSA Resistance to Non-β-Lactams

Since 1961, the incidence of MRSA resistance to β-lactam antibiotics including new-generation cephalosporins is increasing worldwide [64]. MRSA strains become additionally resistant to antibiotics of multiple non-β-lactam classes such as macrolides, aminoglycosides, glycopeptides, oxazolidinone, lipopeptide, pyrimidine/sulfonamide, and others. This is because MRSA strains often harbor genes that convey resistance to antibiotics of multiple non-β-lactam classes. Antibiotic resistance is mediated through several distinct mechanisms, most of which are quite well-understood [65]. Depending on the antimicrobial class, *S. aureus* can utilize different mechanisms to resist the antibiotic effect. In this review, we included the approved non-β-lactam antibiotics currently used for the management of patients with MRSA infections, which are suggested as per the evidence-based guidelines prepared by an Expert Panel of the Infectious Diseases Society of America (IDSA) [29] and United Kingdom (UK) guidelines produced following a review of the published literature (2007–2018) [27]. A summary of the currently used non-β-lactam antibiotic mechanisms of action and molecular bases of resistance in *S. aureus* is presented in Table 1. A better understanding of the molecular basis of antibiotic resistance could help in the development of novel drugs that suppress MRSA in multiple ways and molecular detection methods of antibiotic resistance.

### 4.1. Macrolides (Erythromycin)

Erythromycin, a macrolide antibiotic discovered in 1952, has been used for the treatment of SSTIs caused by MRSA [66]. Macrolide antibiotics inhibit protein synthesis by targeting the bacterial ribosome. They bind to bacterial 23S rRNA on the 50S ribosome subunit and stop protein synthesis by inhibiting the transpeptidation/translocation step of protein synthesis and assembly of the 50S ribosomal subunit [68,69]. Macrolide molecules may also affect the functional properties of the catalytic center of the ribosome, leading to inhibition of translation or a change in the reading frame, resulting in the abnormal synthesis of the polypeptide chain [222]. Macrolides have a broad spectrum of activity against both gram-positive and gram-negative bacteria.

*S. aureus* resistance to erythromycin was first observed in 1955, following its introduction in 1952 [67]. In *S. aureus*, the resistance to macrolides, lincosamides, and streptogramin B (MLS_B_) antibiotics correlates with the resistance to methicillin [223]. MRSA strains showing resistance to MLS_B_ are usually determined by the presence of 23S rRNA methyltransferase encoding *ermA* or *ermC* genes, whereas MSSA strains show resistance by *ermC*, followed by *ermB*, genes [70]. The MLS_B_ resistance can be inducible or constitutive and is not related to the type of *erm* genes.

The main mechanisms leading to macrolide resistance in *S. aureus* are (i) modification of the bacterial ribosome by *erm*-gene-encoded 23S rRNA methyltransferase [70], which leads to a conformational change in the ribosome, thus preventing the binding of macrolides to ribosomal target [31,32]; (ii) active efflux of macrolides from the cell by ATP-binding cassette family (ABC-F) transporters encoded by *msr*A and *msr*B genes, protecting the bacterial ribosome from antibiotic-mediated inhibition [72,73]; (iii) enzymatic inactivation of the macrolides by phosphotransferases (encoded by *mph* genes) [74] and esterase (encoded by *ere* genes), which prevent binding to target site [74]. Indeed, the major genes associated with macrolides resistance in *S. aureus* include *ermA* [31], *ermB*, *ermC* [32], *ermY* [52], *msr*(F) [71], *msrA* [52,72], *msrB*, *ereA*, *ereB*, *mphB*, and *mphC* [52].

### 4.2. Lincosamide (Clindamycin)

Clindamycin, a lincosamide antibiotic approved in 1966, has been used for the treatment of SSTIs caused by CA-MRSA [29]. Clindamycin in MLS_B_ family of antibiotics serves as an alternative to treat both MSSA and MRSA infections due to its excellent pharmacokinetic properties. It inhibits bacterial protein synthesis by binding to the 23S rRNA of the 50S ribosomal subunit and impedes both the assembly of ribosomes and the translation process [75]. It impairs peptide chain initiation and stimulates the dissociation of peptidyl-tRNA from ribosomes.

The widespread use of clindamycin has led to the emergence of resistant strains; the first report of clindamycin resistance in *S. aureus* appeared in 1968 [30]. Resistance to clindamycin generally occurs through ribosomal target site modification mediated by *erm* genes [70], which can be expressed either constitutively (_c_MLS_B_ phenotype) or inducibly (_i_MLS_B_ phenotype) [40,76]. The *ermB, ermC*, and *ermA* genes code for rRNA methylase, which methylates and alters the drug target site 23S rRNA, thus preventing the binding of MLS_B_ antibiotics [77]. Furthermore, resistance to clindamycin occurs through target site modification by *cfr*-gene-encoded rRNA methyltransferase [41], which methylates an adenine residue of the 23S rRNA in the 50S ribosomal subunit, resulting in altered antibiotic binding sites within the ribosome. The emergence of multidrug-resistant CA-MRSA strains carrying plasmid pUSA03 (codes for resistance to clindamycin and mupirocin), predominant among isolates from men who have sex with men (MSM), is on the rise in Boston and San Francisco [224]. Transformation of a large plasmid pSCFS3 that carried the two chloramphenicol- and florfenicol-resistance genes *cfr* and *fexA* from porcine *S. aureus* strain into *S. aureus* RN4220 mediated high-level clindamycin resistance (MIC 256 μg/mL) in addition to chloramphenicol–florfenicol resistance (MICs for both ≥ 128 μg/mL) [42].

### 4.3. Aminoglycosides (Gentamicin)

Gentamicin, an aminoglycoside antibiotic, was approved by the U.S. FDA in 1971 for the treatment of sepsis in newborns, septicemia, and UTI caused by MRSA. Gentamicin acts by binding to the 16S rRNA helix at the mRNA–tRNA decoding center of bacterial 30S ribosome subunit [80,81], thereby causing inaccurate induction and inhibition of translation, disrupting bacterial protein synthesis [82,83,84].

Resistance to aminoglycosides is usually mediated by aminoglycoside-modifying enzyme (AME) AAC(6′)/APH(2″) in *S. aureus* [85]. The AMEs inactivate aminoglycosides by *N*-acetylation (*N*-acetyl transferases, AAC), *O*-phosphorylation (aminoglycoside phosphotransferases, APH), or *O*-adenylation (aminoglycoside nucleotidinyl transferases, ANT) of amino or hydroxyl groups of antibiotics [51]. Gentamicin-resistant *S. aureus* was first reported in 1975 [78,79]. The *aac(6*′*)/aph(2*″*)*-gene-encoded bifunctional AME AAC(6′)/APH(2″) specifies both 6′-acetyltransferase (AAC(6′))- and 2″-phosphotransferase (APH(2″))-aminoglycoside-modifying activities [36,37]. The *aac(6*′*)/aph(2*″) gene is the most prevalent in aminoglycoside-resistant *S. aureus* isolates [86,225].

Arbekacin is a semisynthetic aminoglycoside antibiotic derived from kanamycin. Japanese Pharmaceuticals and Medical Devices Agency (PMDA) approved arbekacin for the treatment of pneumonia caused by MRSA in 1990 [87]. It is not used clinically in the USA. Arbekacin binds to the four nucleotides of the 16S rRNA and one amino acid of protein S12 to interfere with the decoding site around nucleotide 1400 in the 16S rRNA of the 30S ribosome subunit [89]. This site interacts with the wobble base of tRNA, which leads to the misreading of mRNA, such that incorrect amino acids are inserted into the proteins. These error-filled proteins are nonfunctional or even toxic. A single base alteration at site G1126A of the *aac(6*′*)/aph(2*″) gene, resulting in one amino acid substitution S376N in the phosphorylation catalytic motif of AAC(6′)/APH(2″), has been reported to be associated with arbekacin resistance in MRSA strain PRC104 [90]. Moreover, MRSA strain KU5801, which has additional β-lactam-inducible arbekacin resistance, was reported in Japan due to an antagonistic mechanism [91] by which the integration of Tn4001-IS257 hybrid structure that contained the *aac(6*′*)/aph(2*″) gene cointegrated into a region downstream of the *blaZ* gene. A previous study found that all 17 MRSA strains belonging to *coa*-RFLP type M22 possessed the *aac(6*′*)/aph(2*″) gene, but 70.1% were resistant to arbekacin [88], whereas of the 363 MRSA type L21 strains, 5.5% were arbekacin-resistant, despite about half (41.9%) of them carrying the *aac(6*′*)/aph(2*″) gene. This suggests that AAC(6′)/APH(2″) mediate arbekacin resistance, but there is not a single mechanism among MRSA type L21 strains [88]. AAC(6′)/APH(2″) has the capability of 6′-*N*-acetylation and/or 2″-*O*-phosphorylation of arbekacin that contains 6′-NH_2_ and/or 2″-OH [37,92].

### 4.4. Glycopeptides (Vancomycin)

Vancomycin, a glycopeptide antibiotic, was introduced in 1958 for the treatment of severe gram-positive bacterial infections, including MRSA [226]. Vancomycin has long been considered the last-line antibiotic to treat serious infections, such as bacteremia, infective endocarditis, osteomyelitis, meningitis, pneumonia, sepsis, and severe SSTI due to both HA-MRSA and CA-MRSA [29]. Vancomycin inhibits bacterial cell wall synthesis by targeting the D-alanyl-D-alanine (D-Ala-D-Ala) terminus of peptidoglycan [227,228]. It forms hydrogen bonds with D-Ala-D-Ala termini moieties of the peptidoglycan precursor lipid II, resulting in conformational alteration that inhibits the incorporation of *N*-acetylmuramic acid (NAM)- and *N*-acetylglucosamine (NAG)-peptide subunits into the growing peptidoglycan chain, thereby inhibiting bacterial cell wall biosynthesis [95,229]. This alters bacterial membrane integrity and increases its permeability, which ultimately leads to bacterial death [229]. The Clinical and Laboratory Standards Institute (CLSI) has classified *S. aureus* isolates as vancomycin-susceptible *S. aureus* (VSSA; MIC ≤ 2 μg/mL), vancomycin-intermediate *S. aureus* (VISA; MIC of 4–8 μg/mL), and vancomycin-resistant *S. aureus* (VRSA; MIC ≥ 16 μg/mL) [146].

While human infections with MRSA are commonly treated with vancomycin, VISA started appearing in the 1990s [28]. The first *S. aureus* clinical strain with reduced vancomycin susceptibility (MIC 8 μg/mL) was reported in Japan in 1996 [28]. The gradual mutations within genes encoding two-component regulatory systems (TCSs) such as WalKR [104,105,106,107], VraSR [108,109,110], or GraSR [107,109,110,111,112] are predominantly involved in cell wall biosynthesis and are associated with VISA. WalKR is essential for the regulation of cell wall metabolism-associated genes and particularly as a regulator of peptidoglycan synthesis at the time of cross-bridge hydrolysis [230,231,232]. GraSR system is involved in cell envelope modifications through regulation of the *dlt* operon and *mprF/fmtC* genes that are linked to teichoic acid alanylation and alteration of cell wall charge [97]. Mutations within the *graSR* are associated with modified expression of global regulators Rot (repressor of toxins) [233] and accessory gene regulator (Agr) [234], which lead to VISA. Regulator mutation in TCS *walKR* and truncating mutation in proteolytic regulatory gene *clpP* in laboratory-derived VISA strain N315LR5P1 leads to 1 to 2 mg/L raised vancomycin resistance [113]. Furthermore, the mutation in *rpoB* gene encoding the DNA-dependent RNA polymerase β-subunit results in increased cell wall thickness and thereby increased resistance to vancomycin [99,100]. Recently, single-nucleotide polymorphisms (SNPs) in the *capB* (E58K) gene (encoding tyrosine kinase) and *lytN* (I16V) gene (encoding *N*-acetylmuramyl-L-alanine amidase) have been shown to cause increased *S. aureus* resistance to vancomycin in the absence of *van* genes [101].

The occurrence of VRSA infections in clinical settings remains rare [235], which could be due to the antagonistic effects of *mecA* and *vanA* resistance determinants [236]. The development of VRSA strains occurred through horizontal gene transfer (HGT) of the *vanA* gene by transposon Tn*1546* from vancomycin-resistant *Enterococcus faecalis* [237]. The *vanA* gene cluster that encodes D-Ala:D-Lac ligases alters the dipeptide terminus of peptidoglycan precursors from D-Ala-D-Ala to D-Ala-D-lactate (D-Ala-D-Lac) [95,238], which has substantially lower binding affinity for vancomycin and thus fails to inhibit cell wall synthesis in *S. aureus* [102]. The first *vanA*-mediated high-level VRSA (MIC ≥ 32 µg/mL) clinical strain was recovered in Michigan, the USA in 2002 [239]. Furthermore, the first *vanA*-mediated methicillin-resistant VRSA (MIC > 256 µg/mL) strain in Europe was isolated from a patient in Portugal in 2013 [240].

### 4.5. Oxazolidinones (Linezolid)

Linezolid is the first fully synthetic oxazolidinone antibiotic approved by the U.S. FDA in 2000 for the treatment of acute bacterial skin and skin structure infections (ABSSSI), pneumonia, bone and joint infections (BJI), and catheter-related bacteremia caused by gram-positive bacteria with activity against MRSA [120,241]. Linezolid inhibits bacterial protein synthesis by binding to the domain V region of the 23S rRNA of the 50S ribosome subunit and preventing the complex formation with *N*-formyl methionyl–tRNA (tRNA^fMet^), mRNA, and the 30S ribosome subunit [120,122]. The clinical breakpoint of linezolid for MRSA is 8 μg/mL.

MRSA clinical isolates with resistance to linezolid were first reported in 2001 [121]. Further, new oxazolidinone antibiotics such as tedizolid, radezolid, and contezolid with superior efficacy were developed, but MRSA clones with resistance to these antibiotics also evolved [242]. Linezolid resistance in MRSA was due to the acquisition of *cfr* gene, encoding 23S rRNA methyltransferase enzyme [125], which alters adenosine at position 2503 in 23S rRNA in the large ribosomal subunit [126]. A T2500A mutation in the *23S rRNA* gene and loss of a single copy of rRNA has been reported to be associated with linezolid resistance in sequential *S. aureus* isolates [127]. Furthermore, the mutation of domain V of the 23S rRNA [121] and mutation of the ribosomal proteins near the linezolid binding site in the ribosomal peptidyl transferase center [128] results in linezolid resistance in MRSA. Multiple MRSA clones with two mutations in the ribosomal protein uL3 exhibited resistance to linezolid, with a two-fold higher MIC than the clinical breakpoint [243].

### 4.6. Lipopeptides (Daptomycin)

Daptomycin, a cyclic lipopeptide antibiotic approved by the U.S. FDA in 2003, has in vitro bactericidal activity against many gram-positive bacteria. It was approved for the treatment of bacteremia and endocarditis caused by *S. aureus* [244] and has now become the main alternative to vancomycin for serious MRSA infections [245]. Daptomycin exhibits bactericidal activity by targeting membrane phospholipid phosphatidylglycerol as well as bactoprenyl-coupled cell wall precursors such as lipid II in a calcium-dependent manner [246]. In addition, daptomycin affects the localization of cell wall synthesis enzymes like MurG, further interfering with cell wall synthesis [137,138].

Since no resistant breakpoint for daptomycin has been officially established, the term nonsusceptible is used by some researchers over resistant. *S. aureus* strains with MIC ≤ 1 μg/mL are referred as daptomycin-susceptible (DAP-S) [145] and strains with MIC >1 μg/mL as daptomycin-nonsusceptible [146]. The first daptomycin-nonsusceptible *S. aureus* isolates have been reported from a patient treated with daptomycin for bacteremia in Boston in 2004 [135]. Even before the daptomycin approval, the daptomycin-nonsusceptible *S. aureus* mutants were observed after passage through increasing concentrations of daptomycin [247]. Although the development of daptomycin-nonsusceptible *S. aureus* remains rare, there have been steady reports of the emergence of daptomycin-nonsusceptible MRSA strains (MIC of >1 μg/mL) during treatment with daptomycin [248,249,250,251].

The development of daptomycin-nonsusceptible *S. aureus* occurs from the stepwise and multifactorial process that involves cell membrane and cell wall alterations [136,142,252]. The most common resistance mechanism includes the alteration of the surface charge of cells which results in the repulsion of anionic daptomycin molecules [142]. This primarily occurs due to the acquisition of gain-of-function mutations in *mprF* gene encoding a membrane-bound protein MprF called lysyl–phosphatidyl glycerol synthetase [253]. MprF is a bifunctional protein that facilitates both the lysinylation of phosphatidylglycerol, i.e., transfer of negatively charged phosphatidylglycerol to positively charged lysyl–phosphatidylglycerol and the translocation of lysyl–phosphatidylglycerol to the outer leaflet of the membrane [136,144,253,254]. Furthermore, the *dlt* operon regulates the alanylation of wall teichoic acid, and mutation in the *dlt* operon leads to an increase in cell surface positive charge, which reduces the daptomycin susceptibility through charge-mediated repulsion [233,255]. Mutations in various genes including those associated with the cell membrane (*mprF*), cell wall (*dltABCD*), and RNA polymerase subunits (*rpoC* and *rpoB*) [99] have been described to play an important role in daptomycin susceptibility [143,252,256,257]. Daptomycin resistance also occurs due to mutations in *yycH* and *yycI* genes, leading to loss of protein functions and downregulated the WalKR and the downstream players Atl and amidase Sle1, including the autolysin Atl and amidase Sle1 which are essential for cell wall synthesis [141]. Mutations of the TCSs like WalKR, VraSR, or GraSR that directly or indirectly control the transcription of several genes encoding proteins involved in cell wall synthesis and permeability have been also associated with daptomycin susceptibility in *S. aureus* [142,143]. GraSR regulates the expression of genes encoding peptidoglycan hydrolases lysyl–phosphatidylglycerol synthase and flippase, MprF, and the DltABCD system, which modifies teichoic acids with D-alanine [233,258,259].

### 4.7. Fluoroquinolone (Ciprofloxacin)

Ciprofloxacin, a second-generation synthetic antibiotic of the fluoroquinolone class is active against a broad range of gram-positive and gram-negative bacteria. It was approved by the U.S. FDA in 1987 to treat UTIs caused by both MRSA and MSSA. Ciprofloxacin targets bacterial DNA topoisomerase IV and DNA gyrase enzymes which contributes to the relaxation of positive supercoils during DNA replication [176], thus preventing DNA replication and eventually bacterial death [177,178].

The ciprofloxacin-resistant *S. aureus* isolates were described soon after the introduction of the agent into clinical practice [260]. Resistance to fluoroquinolones including ciprofloxacin typically arises as a result of the (i) point mutations in the *grlA*/*grlB* genes encoding the subunits of DNA topoisomerase IV and *gyrA/gyrB* genes encoding the subunits of DNA gyrase [33,261], and (ii) decreased intracellular accumulation and/or active efflux of the drug by membrane-integrated transporter proteins (e.g., NorA) [262]. Challenging the *S. aureus* isolates with the ciprofloxacin elevated the *norA* expression, which potentiates the evolution by increasing the fitness benefit provided by DNA topoisomerase mutations [179]. The initial target mutations occur more frequently in *grlA* gene, whereas additional mutations are found in *gyrA* gene in highly fluoroquinolone-resistant *S. aureus* strains [35]. Mutational changes result in amino acid substitutions in the QRDR of GrlA and GyrA proteins. The GyrA Ser84Leu and GrlA Ser80Phe mutation caused a two-fold increase in minimum bactericidal concentration (MBC) of fluoroquinolone antibiotic DW286 than its corresponding MIC [49,263].

Efflux pump-mediated fluoroquinolone resistance is due to the extrusion of an intracellular drug into the external environment [57,264]. Efflux pumps are usually expressed at low or nondetectable levels but upregulated upon exposure to certain antimicrobials including fluoroquinolones [57,264]. Several efflux pumps have been identified in *S. aureus*, including chromosomally encoded NorA, NorB, NorC, MdeA, MepA, SepA, and SdrM and plasmid-encoded QacA/B, QacG, QacH, QacJ, and Smr [265]. However, the NorA, QacA, and Smr proteins are considered major players in multidrug resistance in *S. aureus* [56,57,61,62]. The chromosomal gene *norA* was first identified in fluoroquinolone-resistant *S. aureus* isolate in Japan in 1986 [266]. NorA is a 388 amino acid protein with 12 transmembrane segments (TMS) which belongs to the Major Facilitator Superfamily (MFS) of secondary transporters [59]. NorA is also involved in resistance to several structurally different compounds including ethidium bromide dye, quaternary ammonium compounds (disinfectants), and other antimicrobials [262,267,268].

### 4.8. Pyrimidines/Sulfonamides (Trimethoprim-Sulfamethoxazole)

Trimethoprim (TMP; 2,4-diamino-5-(3′,4′,5′-trimethoxybenzyl)pyrimidine)/sulfamethoxazole (SMX; 3-(p-aminophenyl sulfonamido)-5-methylisoxazole), also known as co-trimoxazole, is a combination of trimethoprim and sulfamethoxazole class drugs that have been used to treat UTIs, uncomplicated SSTIs, and BJIs caused by CA-MRSA [29,269,270,271]. TMP inhibits bacterial dihydrofolate reductase (DHFR), an enzyme that catalyzes nicotinamide adenine dinucleotide phosphate (NADPH)-dependent conversion of dihydrofolate (DHF) to tetrahydrofolate (THF) [272,273]. TMP is more specific to *S. aureus* DHFR (SaDHFR) than to human DHFR, which particularly inhibits bacterial folic acid synthesis [189]. SMX is a structural analog of para-aminobenzoic acid (PABA), a substrate important for bacterial folic acid synthesis [274]. SMX binds to dihydropteroate synthase (DHPS) which catalyzes the conversion of PABA to dihydropteroate (DHP) during the THF formation [269]. The inhibition of DHPS leads to defective thymidine biosynthesis and thus reduces or inhibits bacterial folic acid synthesis [275]. In general, TMP–SMX has a greater effect as a combination because synergistically they inhibit two consecutive steps in nucleic acid and protein synthesis which are critical for the growth and cell division of bacteria [276].

The widespread use of TMP–SMX for the treatment of staphylococcal infections leads to the emergence of resistance in both MSSA and MRSA [277,278,279]. *S. aureus* shows resistance to TMP–SMX due to the mutation of chromosomal gene *dfrB* encoding SaDHFR and resistance genes that encode variant DHFRs [189,193,194,195], which is the target of TMP [189]. A single amino acid substitution at position 98 (Phe98 to Tyr98) in SaDHFR encoded by *dfrB* led to the intermediate-level TMP resistance (MIC ≤256 mg/L) in *S. aureus* [189]. In contrast, acquired plasmid-borne *dfrA* (also known as *dfrS1*) encoding type S1 DHFR mediates high-level TMP resistance (MIC ≥512 mg/L). The *dfrA*, *dfrB*, *dfrG*, and *dfrK* are important TMP resistance genes known to occur in staphylococci including MRSA [191,192]. *S. aureus* exposed to sub-MIC of TMP-SMX for 14 days resulted in resistant strains due to the F98Y mutation in DHFR encoded by the *dfrB* gene [280]. Mutations in the *dfrB* and *dfrA* have been reported as major determinants of TMP resistance in *S. aureus* clinical isolates [281,282]. The *dfrG* gene encoding the TMP-resistant DHFR enzyme was rarely identified in *S. aureus* clinical isolates [190,283] but mediates TMP resistance [190,195].

### 4.9. Mupirocin

Mupirocin was discovered in 1971 [197] but marketed for clinical use in the UK in 1985 and US in 1988 [198]. It was widely used as a decolonizing agent during the emergence of the CA-MRSA epidemic in the United States in the 1990s. Currently, mupirocin remains the best option for the treatment of MRSA nasal decolonization and SSTI [27,29]. Mupirocin competitively inhibits bacterial isoleucyl t-RNA synthetase, an enzyme encoded by the chromosomal *ileS* gene that promotes the conversion of isoleucine and tRNA to isoleucyl–tRNA, leading to the inhibition of protein and RNA synthesis [201].

Resistance to mupirocin among *S. aureus* clinical isolates was first reported in 1987 [199,200]. The high-level mupirocin resistance (MIC > 500 μg/mL) by *S. aureus* is generally mediated by the expression of plasmid-encoded *mupA* gene [205,206], which encodes an alternate isoleucyl–tRNA synthetase enzyme [208]. Moreover, the *mupB* gene (3102 bp) is also associated with high-level mupirocin resistance in *S. aureus*, which shares 65.5% sequence identity with *mupA* and 45.5% with *ileS* gene [207]. The low-level mupirocin resistance (MIC 8–256 μg/mL) is usually associated with point mutations in the chromosomally encoded *ileS* gene [205], which result in V588F or V631F alterations in the native isoleucyl–tRNA synthetase [202,203,204]. In addition, low-level mupirocin resistance was confirmed by the chromosomal location of *mupA* gene [284] in some *S. aureus* strains from different geographic areas [285].

### 4.10. Fosfomycin

Fosfomycin discovered in 1969 [210], is a phosphonic acid derivative from cultures of Streptomyces spp. It is a broad-spectrum antibiotic used primarily for the treatment of UTIs caused by multidrug-resistant pathogens including MRSA. Fosfomycin interferes with bacterial cell wall synthesis via irreversibly inhibiting the cytosolic enzyme UDP-*N*-acetylglucosamine enolpyruvyl transferase (MurA), which catalyzes the addition of phosphoenolpyruvate to UDP-*N*-acetylglucosamine (UDP-GlcNAc) to form UDP-*N*-acetylmuramic acid (UDP-MurNAc) [211].

Resistance to fosfomycin has been occurring among MRSA clinical isolates either by chromosome-associated defective transport proteins or plasmid-mediated fosfomycin-inactivating enzymes [213]. GlpT and UhpT transporter proteins mediated the uptake of fosfomycin into bacterial cells. Mutations in GlpT (Trp137/Arg) (encoded by *glpT* gene) [213] and UhpT (encoded by *uhpT* gene) [214] reduce the permeability and subsequently prevent fosfomycin from invading the bacterium [212,213]. In addition, the mutation in *murA* gene encoding UDP-*N*-acetylglucosamine enolpyruvyl transferase reduces affinity for fosfomycin [215], conferring various degrees of drug resistance. The fosfomycin-inactivating enzyme thiol-S-transferase (encoded by *fosB* gene) [53,54] catalyzes the inactivation of fosfomycin antibiotic in *S. aureus* [53,54]. FosY protein, a putative bacillithiol transferase (encoded by *fosY* gene present on a genomic island) which shares 65.9–77.5% amino acid identity with FosB and FosD, respectively, confers resistance to fosfomycin in clonal complex 1 (CC1) MRSA isolate from China [217]. The chromosomally encoded major facilitator superfamily efflux transporter Tet38 (encoded by *tet38* gene) of *S. aureus* acts as an efflux transporter of fosfomycin, which is affected by glycerol-3-phosphate (G3P) [216].

### 4.11. Rifampin

Rifampin was discovered in 1965, introduced for clinical therapy in Italy in 1968, and approved in the United States in 1971 [218]. It is used in combination therapy (adjunctive with vancomycin) for the treatment of serious *S. aureus* infections such as endocarditis and BJI [27]. Rifampicin inhibits transcription via binding to the β-subunit of bacterial DNA-dependent RNA polymerase (encoded by *rpoB* gene), leading to suppression of RNA synthesis and subsequent cell death [219,220].

Unfortunately, the frequency of rifampicin-resistant *S. aureus* isolates has increased in recent times [286]. The high-level resistance to rifampin in *S. aureus* is associated with mutations in an extremely conserved region of the RNA polymerase β-subunit called the rifampin resistance-determining region (RRDR) that resulted in an amino acid substitution Gln468/Arg, His481/Tyr, and Arg484/His [43]. Furthermore, the mutation in *rpoB* gene (N967I) causes the amino acid substitution Asn967/Ile in the β-subunit of RNA polymerase [221]. It is also demonstrated that mutations in *rpoB* gene of VISA strain Mu50 are associated with the alteration of vancomycin susceptibility [100]. Mutations within the *rpoB* gene of clinical *S. aureus* isolates are associated with a decrease in daptomycin susceptibility, thus giving a daptomycin non-susceptible (DNS) phenotype [252,287].

## 5. Conclusions

The evolution and spread of MRSA has become a major concern for public health. MRSA strains are intrinsically resistant to almost all β-lactam antibiotics by an acquired *mecA* encoded PBP2a, which can continue peptidoglycan crosslinking in the face of a challenge by β-lactams. Furthermore, MRSA strains are often also resistant to currently used multiple non-β-lactam antibiotics such as erythromycin, clindamycin, gentamicin, linezolid, tetracycline, fusidic acid, ciprofloxacin, ozenoxacin, TMX–SMX, and others. The selective pressure exerted by antibiotics use has led *S. aureus* to develop resistance against one or more antibiotics simultaneously. MRSA can become resistant to non-β-lactam antibiotics through different mechanisms including modification of the antibiotic target, enzymatic inactivation of antibiotics, and/or decreased antibiotic uptake or efflux. This is mainly directed by the acquisition of resistant genes by HGT and genetic mutations owing to the selective pressure of antibiotics. Therefore, revealing molecular determinants that confer resistance to antibiotics in clinical isolates as well as laboratory strains is important for the development of molecular detection methods of antibiotic resistance and designing novel strategies to control MRSA infections.

Antibiotic combination therapy is currently used for treating some MRSA infections, such as ceftaroline plus daptomycin for refractory bacteremia and daptomycin plus rifampicin for biofilm-related infections. However, the continuous emergence of antibiotic-resistant bacteria has highlighted the need for the development of new antibiotics and the identification of novel drug targets to tackle AMR and optimal management of MRSA infections.

## Figures and Tables

**Table 1:** Mechanisms of action of non-β-lactam antibiotics active against *S. aureus* and molecular basis of antibiotic resistance.

Antibiotic Class/ Primary Agent	Approve Year and Use	Primary Target and Mechanisms of Action	Resistance Genes	Mechanism(s) of Resistance	Comments
Macrolides		Protein synthesis			
Erythromycin	1952 [66]. SSTI (Resistance 1955) [67]	Erythromycin binds to bacterial 23S rRNA in the 50S ribosomal subunit and stops protein synthesis by inhibiting the transpeptidation/translocation step of protein synthesis and assembly of the 50S ribosomal subunit [68,69]. The target site for macrolides is nucleotides A2058 and A2059 located in the V region of 23S rRNA and, rarely, nucleotide A752 located in domain II [70].	*ermA* [31], *ermB*, *ermC* [32], *ermY* [52], *msr*(F) [71], *msrA* [72], *msrB*, *ereA*, *ereB,* *mphB, mphC* [52]	(i) Modification of the bacterial ribosome by 23S rRNA methyltransferase (encoded by *erm* genes) prevents the binding of erythromycin to ribosomal target [31,32]. (ii) Active efflux of macrolides from cells by ATP-binding-cassette family (ABC-F) transporters (encoded by *msrA* and *msrB* genes) protects ribosomes from inhibition [72,73]. (iii) Enzymatic hydrolysis of 14- and 15-membered lactone ring of macrolides by esterase (encoded by *ere* genes) prevents its binding to the antibiotic target site [74]. (iv) Phosphotransferases (encoded by *mph* genes) introduce phosphate to the 2′-hydroxyl group of the 14-, 15-, and 16-membered lactone rings of macrolides amino sugar, which interferes with the interaction of the antibiotic with nucleotide A2058 [52].	Modification of the bacterial ribosome and active efflux from the bacterial cell are important mechanisms of macrolide resistance in *S. aureus*.
Lincosamides		Protein synthesis			
Clindamycin	Discovered in 1966. SSTI caused by CA-MRSA [29] (Resistance 1968) [30]	Clindamycin binds to bacterial 23S rRNA in the 50S ribosomal subunit and impedes both the assembly of ribosomes and the translation process [75].	*ermA*, *ermB*, *ermC* [76] *cfr* [41,42]	(i) The rRNA methylase (encoded by *erm* genes) methylates an adenosine nucleotide within the peptidyl transferase center, resulting in the C-8 methylation of A2503 (m^8^A2503) [77]. (ii) The acquired *cfr* gene encoded rRNA methyltransferase methylates an adenine residue of the 23S rRNA in the 50S ribosomal subunit [41], resulting in altered antibiotic binding sites within the ribosome.	
Aminoglycosides		Protein synthesis			
Gentamicin	U.S. FDA 1971. Bacterial meningitis, sepsis of newborns, septicemia, UTI (Resistance 1975) [78,79]	Gentamicin binds to the A-site on the 16S rRNA helix at the mRNA-tRNA decoding center of bacterial 30S ribosome subunit [80,81], causing the inhibition and inaccurate induction of translation, disrupting protein synthesis [82,83,84].	*aac(6*′*)/aph(2*″) *aadD* (AG *O*-adenyltransferase) [85] *ant*(4′) (AG *O*-nucleotidyltransferase(4′)) *ant*(9) (*AG O*-nucleotidyltransferase(9))	The bifunctional AMEs inactivate aminoglycosides by acetylating, phosphorylating, or adenylating amino or hydroxyl groups [51,85] Gentamicin, tobramycin and kanamycin resistance is generally mediated by a bifunctional AME AAC(6′)-APH(2″) (encoded by *aac(6*′*)/aph(2*″*)* gene*)* that specifies 6′-acetyltransferase [AAC(6′)] and/or 2″-phosphotransferase [APH(2″)] aminoglycoside modifying activities [36,37].	The *aac(6*′*)/aph(2*″*)* gene is most prevalent in aminoglycoside resistant *S. aureus* [86].
Arbekacin (not used clinically in the U.S.)	Japanese PMDA 1990 [87]. Pneumonia and sepsis due to MRSA. (Resistance 1979) [88]	Arbekacin binds to both 50S and the 30S ribosomal subunits, resulting in codon misreading and inhibition of translation [89].	*aac*(*6*′)-*aph*(*2*″) [88,90]	(i) A single base alteration (G1126A) of *aac(6*′*)/aph(2*″*)* gene resulted in one amino acid substitution S376N in AAC(6′)/APH(2″), which leads to arbekacin resistance in MRSA strain PRC104 [90]. (ii) β-lactam-inducible arbekacin resistance was reported in MRSA strain by the integration of Tn4001-IS257 hybrid structure containing *aac(6*′*)/aph(2*″*)* gene cointegrated into a region downstream of *blaZ* gene [91].(iii) The AAC(6′)/APH(2″) modify arbekacin by 6′-*N*-acetylation and/or 2″-*O*-phosphorylation of AGs that contain 6′-NH_2_ and/or 2″-OH [37,92].	Arbekacin is not inactivated by AMEs (3′)(APH), (4′)(AAD), or AAD(2″) and has a weak affinity to (6′-IV) (AAC) [93].
Glycopeptides		Cell wall synthesis			
Vancomycin	1958. Bacteremia, infective endocarditis, osteomyelitis, meningitis, pneumonia, sepsis, and complicated SSTI due to HA-MRSA and CA-MRSA [29]. (Resistance VISA in 1996 [28] and VRSA in 2002 [94])	Vancomycin bind to D-Ala-D-Ala termini moieties of Lipid II precursor of peptidoglycan through a series of hydrogen bonds, leading to conformational alteration that prevents incorporation of NAM- and NAG-peptide subunits to the growing peptidoglycan chain and consequent transpeptidation [95,96,97]. This alters membrane integrity and increases permeability, leading to bacterial death.	*vanA* [97,98] Mutations in *walKR, vraSR*, *graSR,* and *clpP* Mutation in *rpoB* [99,100] SNPs in *capB* (E58K) and *lytN* (I16V) gene [101]	(i) VRSA: The Tn*1546*-borne *vanA* gene cluster encodes 9 proteins (D-Ala:D-Lac ligases) that modify D-Ala-D-Ala termini of peptidoglycan chains to D-Ala-D-Lactate, thereby inhibiting target binding by vancomycin [102,103]. (ii) VISA: Mutations in TCSs like essential *WalKR* [104,105,106,107], *VraSR* [108,109,110], and *GraSR* [107,109,110,111,112] affect cell wall biosynthesis, resulting in reduced susceptibility to vancomycin. (iii) Mutation in *rpoB* (encoding RNA polymerase subunit B) [99,100]. (iv) Mutation in TCS *walKR* and proteolytic regulatory gene *clpP* leads to raised vancomycin resistance in laboratory VISA strain N315LR5P1 [113]. (v) SNPs in *capB* (E58K) gene (encoding tyrosine kinase) and *lytN* (I16V) gene (encoding *N*-acetylmuramyl-L-alanine amidase) cause increased *S. aureus* resistance to vancomycin in the absence of *van* genes [101].	VRSA is mediated by the *vanA* gene cluster, which is transferred from vancomycin-resistant Enterococcus [114].
Teicoplanin (formerly known as teichomycin A2)	1988. Approved in Europe for SSTI, pneumonia, and sepsis [115]. Never approved for use in the U.S. (Resistance 2000) [116]	Teicoplanin inhibits peptidoglycan polymerization, leading to the inhibition of bacterial cell-wall synthesis.	*tcaRAB* [117,118], *tcaA* [119]	(i) The *tcaRAB* operon may be involved in increased teicoplanin resistance in *S. aureus* [118]. (ii) Mutation in *tcaRAB* may influence the transcription of the cell wall biosynthesis gene and may contribute to increasing teicoplanin resistance [117]. (iii) The *tcaA* gene within *tcaRAB* plays a relevant role in teicoplanin resistance in *S. aureus* clinical isolates [119].	BSAC recommended breakpoint for teicoplanin are susceptible (MIC ≤ 2 mg/L) and resistant (MIC > 2 mg/L).
Oxazolidinones		Protein synthesis			
Linezolid	U.S. FDA 2000. ABSSSI, pneumonia, BJI, catheter- related bacteremia [120] (Resistance 2001) [121]	Linezolid binds to bacterial 23S rRNA in the 50S ribosome subunit, thereby preventing the formation of functional 70S ribosomal initiation complex with 30S subunit, mRNA, initiation factors, and *N*-formylmethionyl-tRNA (tRNA^fMet^) [122].	*cfr* [123] Mutations in *23S rRNA* [121,124]	(i) Acquisition of *cfr* gene encoding 23S rRNA methyltransferase [125], which modifies adenosine at position 2503 in 23S rRNA in the large ribosomal subunit [126]. (ii) The T2500A mutation in the 23S rRNA gene and loss of a single copy of rRNA [127]. (iii) Mutations G2576T, G2576T, G2447T in domain V of 23S rRNA [121,124] and amino acid changes in ribosomal proteins L3 and L4 [128] lead to conformational changes in the ribosome.	
Tedizolid	U.S. FDA 2014; E.U. EMA 2015.ABSSSI and pneumonia	Tedizolid binds to 23S rRNA in the 50S ribosome subunit and prevents the formation of 70S ribosomal initial complex, resulting in inhibition of bacterial protein synthesis [129,130].	*cfr* *rplC*, *rplD, rplV*, *rpoB* [131]	(i) Mutations in domain V region of 23S rRNA target confer resistance to tedizolid. (ii) Mutations in ribosomal proteins L3, L4, and L22 (encoded by *rplC*, *rplD*, and *rplV* genes*,* respectively) and the 23S rRNA target [132]. (iii) Mutation in *rpoB* corresponding to amino acid substitution D449N [131].	Mutation in L3, L4, and L22 also mediate PhLOPSa (phenicol, lincosamide, oxazolidinone, pleuromutilin, and streptogramin A) resistance.
Contezolid	NMPA of China 2021 [133]. Complicated SSTI, ABSSSI (Resistance 2021) [134].	Contezolid binds to the 23S rRNA region adjacent to the peptidyl transferase center of the 50S ribosomal subunit and prevents the formation of a functional 70S initiation complex, thereby interfering with bacterial protein synthesis.	*cfr, optrA*	Contezolid exhibited limited activity against strains with linezolid resistance genes *cfr* and *optrA* [134].	Contezolid has reduced hematologic toxicity compared to linezolid
Lipopeptides		Cell wall synthesis Cell membrane			
Daptomycin	U.S. FDA 2003. Bacteremia, ABSSSI (Nonsusceptible 2004) [135]	Daptomycin complexes with Ca^2+^ to form oligomers that insert into bacterial membranes, resulting in depolarization, permeabilization, leakage of ions, and ultimately bacterial death [136]. Daptomycin disrupts the localization of cell wall synthesis enzymes such as MurG, further interfering with cell wall synthesis [137,138].	*mprF, dltA* [139,140]*, yycH, yycI* [141], *rpoB* [99]*, walKR,* *vraSR*, *graSR* [142,143]	(i) Alteration of the surface charge of cells due to mutation in *mprF* gene (encoding phosphatidylglycerol lysyltransferase) which leads to lysinylation of PG and translocation of lysyl-PG [144]. (ii) Mutation in TCSs *walKR,* *vraSR*, and *graSR* which are involved in cell wall synthesis and permeability are associated with daptomycin susceptibility in *S. aureus* [142,143]. (iii) Mutation in *rpoB* gene (encoding RNA polymerase) confers dual heteroresistance to daptomycin and vancomycin [99]. (iv) Mutations in *yycH* and *yycI* genes lead to the loss of protein functions essential for cell wall synthesis [141]. (v) *dltA* gene overexpression leads to electrostatic repulsion and indirectly reduces autolysin, resulting in daptomycin nonsusceptibility [139,140].	*S. aureus* strains with MIC ≤ 1 μg/mL are referred as daptomycin-susceptible (DAP-S) [145] and MIC >1 μg/mL as daptomycin-non susceptible [146].
Lipoglycopeptides		Cell wall synthesis			
Telavancin (derivative of vancomycin. Addition of the hydrophobic side chain and hydrophilic group results in enhanced activity [147].	U.S. FDA 2009 and 2013 [148]. Complicated SSTI, pneumonia, BJI, ABSSSI, bacteremia [149].	Telavancin inhibits cell wall biosynthesis by binding to late-stage peptidoglycan synthesis, like vancomycin. Additionally, it depolarizes the bacterial cell membrane and disrupts its functional integrity [150].	-	The *vanA*-mediated telavancin resistance is rare in MRSA [151].	
Tetracyclines		Protein synthesis			
Tetracycline	1948 [152] SSTI (Resistance 1953) [44]	Tetracycline binds to bacterial 30S ribosomal subunit and prevents the aminoacyl tRNA from binding to A site of the rRNA, resulting in inhibition of translation. To some extent, it also binds to the bacterial 50S ribosomal subunit [44,153,154].	*tetM, tetO*, *tetK* [155]*, tetS/M, tetA*	(i) Ribosomal protection: the *tetM* and *tetO* genes encode enzymes that destabilize the interaction between tetracyclines and their cellular target ribosome [44,45]. (ii) Active efflux: the *tetK* gene encodes efflux protein that couples the tetracycline with proton motive force to pump it out from the cell against the concentration gradient [44,155].	The *tetK* gene is normally found *in S. aureus*.
Doxycycline	U.S. FDA 1967 [156,157]. UTI, SSTI [27]	Doxycycline inhibits bacterial protein synthesis by preventing the association of aminoacyl tRNA with the ribosome, an MoA similar to tetracycline.	*tetK* [158,159]	Active efflux by *tetK* encoded efflux [158,159].	
Tigecycline	U.S. FDA 2005.ABSSSI, pneumonia	Tigecycline inhibits protein synthesis, an MoA similar to tetracycline but with enhanced binding.	*tetM, tetO, tetX*	The oxygen-dependent destruction of tigecycline is catalyzed by the enzyme TetX [160,161,162].	Tigecycline retains activity against both *tetM* and *tetO.*
Omadacycline (derived from tetracycline) [163]	U.S. FDA 2018.ABSSSI, SSTI [164], pneumonia (CA-associated)	Omadacycline binds to bacterial 30S ribosomal subunit and inhibits protein synthesis, an MoA similar to tetracycline with enhanced binding like tigecycline [165].	-	Resistance mechanism not reported.	Unaffected by the presence of *tetK* active efflux gene and ribosomal protection *tetM* or *tetO* gene [166,167].
Fusidane		Protein synthesis			
Fusidic acid	1962. ABSSSI	Fusidic acid binds to elongation factor G (EF-G) on the ribosome, thereby preventing the release of EF-G-guanosine diphosphate complex and delaying bacterial protein synthesis by inhibiting the next stage in translation [168,169].	*fusA* [170]*, fusB* [171,172], *fusc, fusD*	(i) Mutations in chromosomal *fusA* (encoding ribosomal translocase and translation elongation factor EF-G) [170] or *fusE* genes confer high-level resistance to fusidic acid. (ii) Mutation in acquired genes *fusB* (encoding an inducible protein that protects an in vitro translation) [171,172] and *fusD* genes mediate low-level resistance. These mutations affect the elongation factor EF-6.	The *fusc* and *fusD* are homologs of *fusB* [173].
Pleuromutilin		Protein synthesis			
Retapamulin	U.S. FDA 2007. Impetigo [174]	Retapamulin binds to domain V of 23S rRNA on the 50S ribosome subunit, thereby blocking peptide formation directly by interfering with substrate binding.	*23S rRNA*	Resistance to retapamulin occurs due to mutations in the genes encoding 23S rRNA methyltransferase.	Retapamulin is a semisynthetic derivative of pleuromutilin
Fluoroquinolones		DNA replication			[46,175]
Ciprofloxacin (2^nd^-generation fluoroquinolone)	U.S. FDA 1987.UTI	Ciprofloxacin target bacterial DNA topoisomerase IV and DNA gyrase, thus preventing it from supercoiling the bacterial DNA [176], which leads to inhibition of DNA replication [177,178].	*gyrA* [33]*, grlA* [33]*, flqA* (formerly *ofx/cfx*) [35], *norA* [58,179]	(i) Mutation in the genes *grlA* (encoding DNA topoisomerase IV subunit A) [33,34,35,46], *gyrA* (encoding DNA gyrase subunit A) [33,34,35], and *flqA* (linked to DNA topoisomerase IV) [35]. (ii) Mutations in the gene *norA* (encoding a membrane-associated active efflux pump NorA) [58,180].	Elevated *norA* expression potentiates evolution by increasing the fitness benefit provided by a mutation in DNA topoisomerase [179].
Levofloxacin	U.S. FDA 1996. RTI, UTI, SSTI	Levofloxacin inhibits bacterial DNA replication, an MoA similar to ciprofloxacin.	*gyrA, grlA*	(i) Mutation in the genes *grlA* and *gyrA* [181]. (ii) Mutations in the gene *norA* [180].	
Delafloxacin (previously referred to as ABT-492) [182]	U.S. FDA 2017 [183]; E.U. EMA 2019. SSTI, ABSSSI (Resistance 2017) [184]	Delafloxacin inhibits bacterial DNA replication by blocking both DNA topoisomerase IV and DNA gyrase, an MoA similar to ciprofloxacin [182].	*grlA*	Point mutations in the *grlA* [185,186].	Delafloxacin is not active substrate for *S. aureus* efflux pumps [185].
Quinolones		DNA replication			
Ozenoxacin (topical quinolone without fluorine at C6-position)	U.S. FDA 2017.Japanese PMDA 2016 [187]. SSTI (impetigo) caused by MRSA	Ozenoxacin inhibits bacterial DNA replication by dual-targeting activity against DNA topoisomerase IV and DNA gyrase [35].	*grlA, grlB*	Mutations in QRDR regions of *grlA* and *gyrA* are the primary cause of decreased susceptibility to ozenoxacin [35].	Low MIC of ozenoxacin was observed for MSSA and MRSA strains with reduced susceptibility to nadifloxacin [187].
Pyrimidine/ Sulfonamide		Folate synthesis (DNA synthesis and protein synthesis)			
Trimethoprim–Sulfamethoxazole (TMP-SMX)	UTI, SSTI, and BJI due to CA-MRSA [29]	TMP binds and inhibits the dihydrofolate reductase, thereby preventing the conversion of dihydrofolic acid (DHF) to tetrahydrofolic acid (THF) [188]. THF is an essential precursor of the thymidine synthesis pathway and interference with this pathway results in inhibition of bacterial DNA synthesis. SMX inhibits bacterial dihydropteroate synthase, an enzyme involved upstream in the thymidine synthesis pathway, resulting in the inhibition of folic acid biosynthesis [188].	*dfrA*, *dfrB* [189], *dfrD* [189], *dfrG* [190], *dfrK,* *dfrS1* [191,192]	(i) The acquisition of *dfrA* gene (encoding DHFR) and mutation of the chromosomal *dfrB* gene (encoding SaDHFR) are considered key determinants of TMP-SMX resistance [189,193,194,195]. (ii) Point mutation in the *dfrB* gene resulted in a single amino acid substitution Phe98Tyr of SaDHFR, which was associated with TMP-SMX resistance in *S. aureus* [189]. (iii) Transposon-located *dfrA* gene mediates TMP resistance [194,196]. (iv) The *dfrG* gene (encoding DHFR) mainly mediates the TMP resistance in *S. aureus* clinical isolates [190,195].	
Other classes					
Mupirocin (previously pseudomonic acid)	Discovered in 1971 [197] while marketed for clinical use in the UK in 1985 and US in 1988 [198]. SSTI, nasal carriage of *S. aureus* (Resistance 1987) [199,200].	Mupirocin binds to bacterial isoleucyl transfer RNA (tRNA) synthetase, leading to depletion of isoleucyl–tRNA and accumulation of the corresponding uncharged tRNA. This results in the inhibition of protein and RNA synthesis [201].	*ileS* [202,203,204]*, mupA* [205,206], and *mupB* [207]	(i) Mutations in the chromosomal *ileS* gene (encoding native isoleucyl t-RNA synthetase) result in V588F or V631F alterations [202,203,204], which lead to low-level mupirocin resistance [205]. (ii) Acquisition of the plasmid-encoded *mupA* gene (encoding eukaryotic-like isoleucyl–tRNA synthetase variant) [208] confers high-level resistance to mupirocin [205,206]. (iii) Acquisition of the plasmid-encoded *mupB* gene (encoding eukaryotic-like isoleucyl–tRNA synthetase variant) confers high-level resistance to mupirocin [207].	Low-level mupirocin resistance (MIC 8–256 μg/mL) and high-level resistance (MIC ≥ 512 μg/mL) [209].
Fosfomycin	Discovered in 1969 [210]. UTI	Fosfomycin deactivates the enzyme UDP-*N*-acetylglucosamine enolpyruvyl transferase (MurA) and catalyzes the addition of phosphoenolpyruvate to UDP-*N*-acetylglucosamine (UDP-GlcNAc) to form UDP-*N*-acetylmuramic acid (UDP-MurNAc), thereby inhibiting bacterial cell-wall synthesis [211].	*fosB* [54], *glpT* and *uhpT* [212,213,214], *murA* [213,215] *tet38* [216], *fosY* [217]	(i) Thiol-S-transferase (encoded by *fosB* gene) catalyzes the inactivation of fosfomycin [53,54]. (ii) Mutations in fosfomycin uptake transporter proteins GlpT (Trp137/Arg) (encoded by *glpT* gene) [213] and UhpT (encoded by *uhpT* genes) [214] reduce the permeability and subsequently prevent fosfomycin from invading the bacterium [212,213]. (iii) Mutation in target enzyme UDP-*N*-acetylglucosamine enolpyruvyl transferase (encoded by *murA* gene) reduces its affinity for fosfomycin [215]. (iv) The major facilitator superfamily efflux transporter Tet38 (encoded by *tet38* gene) contributes to fosfomycin resistance [216]. (v) FosY protein, a putative bacillithiol transferase enzyme (encoded by *fosY* gene) confers resistance to fosfomycin in CC1 *S. aureus* [217].	
Rifampin	Discovered in 1965, introduced for therapy in Italy in 1968, and approved in the United States in 1971 [218]. Endocarditis; BJI [27].	Rifampin inhibits transcription (RNA synthesis) by binding to the β-subunit of the bacterial DNA-dependent RNA polymerase [219,220].	*rpoB* [43,221]	(i) Mutations in the RRDR region of *rpoB* gene (encoding RNA polymerase) resulted in amino acid substitutions of Gln468/Arg, His481/Tyr, and Arg484/His and are associated with high-level resistance to rifampicin [43]. (ii) Mutation in the *rpoB* (N967I) gene causes the substitution Asn967/Ile in the β-subunit of RNA polymerase [221].	CLSI breakpoint of rifampicin susceptibility is ≤1 μg/mL [146].

–: not studied or reported, AAC: aminoglycoside acetyltransferase, ABSSSI: acute bacterial skin and skin structure infection, AG: aminoglycoside, AMEs: aminoglycoside-modifying enzymes, ANT: aminoglycoside nucleotidyltransferase, APH: aminoglycoside phosphotransferase, BJI: bone and joint infections, BSAC: British Society for Antimicrobial Chemotherapy, CLSI: Clinical and Laboratory Standards Institute, E.U. EMA: European Union European Medicine Agency, Japanese PMDA: Japanese Pharmaceutical and Medical Devices Agency, MRSA: methicillin-resistant *S. aureus*, MSSA: methicillin-sensitive *S. aureus*, PG: peptidoglycan, QRDR: quinolone-resistance-determining region, RRDR: rifampin-resistance-determining region, rRNA: ribosomal RNA, SaPI: *S. aureus* pathogenicity island, SMX: sulfamethoxazole, SSTI: skin and soft tissue infections, TCSs: two-component regulatory systems, TMP: trimethoprim, U.S. FDA: U.S. Food and Drug Administration, UTI: urinary tract infection, VISA: vancomycin intermediate-resistant *S. aureus*, VRSA: vancomycin-resistant *S. aureus.*

## Data Availability

Not applicable.
